# Clinical Characteristics of Endogenous Cushing's Syndrome at a Medical Center in Southern Taiwan

**DOI:** 10.1155/2013/685375

**Published:** 2013-08-22

**Authors:** Shih-Chen Tung, Pei-Wen Wang, Rue-Tsuan Liu, Jung-Fu Chen, Ching-Jung Hsieh, Ming-Chun Kuo, Joseph W. Yang, Wei-Ching Lee, Min-Hsiung Cheng, Tao-Chen Lee

**Affiliations:** ^1^Division of Endocrinology and Metabolism, Department of Internal Medicine, Kaohsiung Chang Gung Memorial Hospital and Chang Gung University College of Medicine, 123 Ta-Pei Road, Niao-Sung District, Kaohsiung City 83301, Taiwan; ^2^Division of Urology, Department of Surgery, Kaohsiung Chang Gung Memorial Hospital and Chang Gung University College of Medicine, 123 Ta-Pei Road, Niao-Sung District, Kaohsiung City 83301, Taiwan; ^3^Division of Neurosurgery, Department of Surgery, Kaohsiung Chang Gung Memorial Hospital and Chang Gung University College of Medicine, 123 Ta-Pei Road, Niao-Sung District, Kaohsiung City 83301, Taiwan

## Abstract

From January 1987 to December 2011, over a total of 25 years, 84 patients with Cushing's syndrome (CS) were identified at a medical center in southern Taiwan. We observed a higher incidence of ACTH-independent CS (75%) than ACTH-dependent CS (25%). A higher incidence of adrenocortical adenoma (58.3%) than Cushing's disease (CD, 21.4%) was also found. The sensitivity of the definitive diagnostic tests for CS, including loss of plasma cortisol circadian rhythm, a baseline 24 h urinary free cortisol (UFC) value >80 **μ**g, and overnight and 2-day low-dose dexamethasone suppression test, was between 94.4% and 100%. For the 2-day high-dose dexamethasone suppression test for the differential diagnosis of CD, the sensitivity of 0800 h plasma cortisol and 24 h UFC was 44.4% and 85.7%, respectively. For the differential diagnosis of adrenal CS, the sensitivities of the 0800 h plasma cortisol and 24 h UFC were 95.5% and 88.9%, respectively. In patients with ACTH-independent CS and ACTH-dependent CS, the baseline plasma ACTH levels were all below 29 pg/mL and above 37 pg/mL, respectively. The postsurgical hospitalization stay following retroperitoneoscopic adrenalectomy was shorter than that observed for transabdominal adrenalectomy (4.3 ± 1.6 versus 8.8 ± 3.7 days, *P* < 0.001). It was easy to develop retroperitoneal and peritoneal seeding of adrenocortical carcinoma via laparoscopic adrenalectomy.

## 1. Introduction

The definitive diagnosis of endogenous Cushing's syndrome (CS) is a challenge in clinical endocrinology. Biochemical confirmation of CS relies upon the measurement of urinary free cortisol (UFC) over a 24 h period, low dose dexamethasone suppression test (LDDST), and plasma cortisol circadian rhythm assessment [1, 2]. Biochemical tests for the differential diagnosis of CS include baseline plasma adrenocorticotropic hormone (ACTH) measurement, high-dose dexamethasone suppression test (HDDST), and corticotropin-releasing hormone (CRH) stimulation test [3–5].

CS is classified as either ACTH dependent or ACTH independent. In Caucasians, the most common form of the syndrome is ACTH dependent (80–85%). Cushing's disease (CD, excessive secretion of corticotropin by pituitary corticotroph tumors), accounts for 60–80% of cases in most series [3, 5–7]. Ectopic ACTH CS represents approximately 12–16% of cases [5–7]. ACTH-independent CS (15–20%) results from excessive secretion of cortisol by adrenocortical tumors, classified as either adenoma (10%) or carcinoma (8%), by primary pigmented nodular adrenocortical disease (PPNAD, 1%) or by ACTH-independent bilateral macronodular adrenocortical hyperplasia (AIMAH, <1%) [3, 6–8].

In this study, we analyzed the clinical characteristics of CS at a medical center in southern Taiwan and observed a different population distribution compared to Western countries for ACTH-independent CS and ACTH-dependent CS.

## 2. Materials and Methods

### 2.1. Patients

From January 1987 to December 2011, over a total of 25 years, 84 patients with CS were confirmed by clinical manifestation, plasma and/or urinary biochemical laboratory tests, and surgical pathology. These patients were divided into (a) those with ACTH-independent (adrenal) CS, including adrenocortical adenomas and carcinomas, PPNAD, and AIMAH and (b) those with ACTH-dependent CS, including CD and ectopic ACTH syndrome (EAS). Patients who were suspected of endogenous CS without surgical intervention were excluded. Adrenal incidentalomas were not included in this study.

### 2.2. Study Design

Laboratory tests of hypothalamic-pituitary-adrenal function in CS included measurement of baseline morning (0800 h to 0900 h) and afternoon (1600 h to 2200 h) plasma cortisol levels, baseline 24 h UFC, loss of plasma cortisol circadian rhythm (i.e., baseline afternoon plasma cortisol level greater than 7.5 *μ*g/dL and greater than 50% of baseline morning plasma cortisol level), nonsuppression of morning 0800 h plasma cortisol level (i.e., greater than 5 *μ*g/dL) after administration of 1 mg dexamethasone orally at 2300 h (overnight low-dose dexamethasone suppression test, overnight LDDST), and nonsuppression of 24 h UFC (>122 *μ*g) or nonsuppression of morning 0800 h plasma cortisol level (>5 *μ*g/dL) 48 h after 0.5 mg dexamethasone administered orally every 6 h for 2 days (2-day LDDST). Baseline morning (0800 h to 0900 h) plasma ACTH levels were also measured. For the 2-day high-dose dexamethasone suppression test (2-day HDDST, 2 mg dexamethasone administered orally every 6 h for 2 days), a plasma cortisol level greater than 50% of the baseline plasma cortisol level or a 24 h UFC level greater than 50% of the baseline 24 h UFC level was indicative of nonsuppression.

### 2.3. Assays and Statistical Analysis

Plasma cortisol and UFC levels were measured by radioimmunoassay (RIA). Plasma ACTH was measured either by RIA or by immunoradiometric assay. Normal ranges for baseline plasma cortisol are 7.00–25.00 *μ*g/dL in the morning and 2.00–9.00 *μ*g/dL in the afternoon. Baseline plasma ACTH levels in the morning are 9–52 pg/mL. Baseline 24 h UFC values are 34–122 *μ*g/24 h. Data are expressed as the mean ± the standard deviation (SD). Statistical analyses were performed using SPSS 12.0 (SPSS, Chicago, IL, USA). A *P* < 0.05 is reported as statistically significant.

## 3. Results

### 3.1. Clinical Features

The patient distribution and demographic data for 84 patients with CS are presented in Table 1. The mean age of those with ACTH-independent CS and ACTH-dependent CS at the time of surgery was 37.5 ± 14.2 and 40.9 ± 16.1 years old, respectively. A larger proportion of our patients had ACTH-independent CS versus ACTH-dependent CS (75% versus 25%). Forty-nine of the 84 patients (58.3%) had adrenocortical adenoma, whereas 18 (21.4%) had CD. More women than men had adrenocortical adenoma as well as CD (Table 1). Of the 54 patients with unilateral adrenocortical adenoma or carcinoma, the left side (33 cases) was affected more often than the right one (21 cases).

There were seven cases of adrenocortical carcinoma. One patient with this disease had a tumor 3.3 cm in diameter, as visualized by abdominal computed tomography. After laparoscopic adrenalectomy, the patient developed retroperitoneal and peritoneal seeding.

### 3.2. Definitive Diagnosis of Cushing's Syndrome (Table 2)

In all patients with CS, baseline morning plasma cortisol levels were often within the normal range (60%, 48 out of 80 cases) but mostly lacked circadian rhythm (95.9% of sensitivity). The mean baseline plasma cortisol levels of all CS patients at 0800 h and 1600 h–2200 h were similar (24.6 ± 10.5 versus 22.3 ± 10.0 *μ*g/dL; *P* = 0.155). The sensitivity of the baseline 24 h UFC values was 88.5% using a cut-off value greater than 122 *μ*g/24 h. If the cut-off value for the baseline 24 h UFC was set at greater than 80 *μ*g/24 h, the sensitivity increased to 96.2%. The sensitivity of overnight and 2-day LDDST using the criteria of plasma cortisol levels greater than 5 *μ*g/dL (indicative of nonsuppression) was 98.2% and 100%, respectively. The sensitivity of the 2-day LDDST using the criterion of a 24 h UFC value greater than 122 *μ*g/day (indicative of nonsuppression) was 94.4%. Five patients in the ACTH-independent CS group had a baseline 24 h UFC level <122 *μ*g/24 h, with individual values of 32.5, 88, 90, 94, and 112 *μ*g/24 h. One patient with ACTH-dependent CS also had a very low baseline 24 h UFC level (10.7 *μ*g/24 h).

### 3.3. Differential Diagnosis of Cushing's Syndrome

For the 2-day HDDST, the sensitivities of 0800 h plasma cortisol levels and 24 h UFC levels using the criteria of more than 50% suppression relative to the baseline values (i.e., suppression) for patients with CD were 44.4% and 85.7%, respectively. The sensitivities of 0800 h plasma cortisol levels and 24 h UFC levels using a cut-off of greater than 50% of baseline levels (i.e., nonsuppression) for patients with adrenal CS were 95.5% and 88.9%, respectively (Table 2).

Baseline morning plasma ACTH levels were measured in 62 patients with CS (Table 3). The levels were all below 29 pg/mL in the 43 patients with ACTH-independent CS. Five (11.6%) of those adrenal CS patients had plasma ACTH levels between 20 and 28.7 pg/mL (Table 3). Baseline plasma ACTH levels were between 37.4 and 328 pg/mL in the 16 patients with CD. Three (18.8%) of these patients were found to have levels within the normal range (37.4, 45.8, and 46.8 pg/mL) (Table 3). Baseline plasma ACTH levels in three patients with EAS (small cell lung carcinoma, meningioma, and carcinoid tumor) were 427, 725, and 318 pg/mL, respectively.

### 3.4. Retroperitoneoscopic versus Traditional Transabdominal Adrenalectomy for Adrenal Cushing's Syndrome

Of the sixty-three patients who had adrenal CS, four (3 PPNAD patients and 1 patient with bilateral adrenocortical adenoma) received 2 surgical operations each on different dates [9–12], for a total number of 67 operations performed. Twenty-four cases with adrenal nodules (including adenoma, carcinoma, AIMAH, and PPNAD) were treated with retroperitoneoscopic adrenalectomy. These patients had a mean postsurgical hospitalization stay of 4.3 ± 1.6 days (mean ± SD). The other forty-three (including adenoma, carcinoma, AIMAH, and PPNAD) were treated with traditional transabdominal adrenalectomy. Their postsurgical hospitalization stay was 8.8 ± 3.7 days. The difference between the two groups with regard to postsurgical hospital stay was significant (*P* value < 0.001).

## 4. Discussion

In our study, the incidence of ACTH-independent CS (75%) was markedly higher than that of ACTH-dependent CS (25%), and the incidence of adrenocortical adenoma (58.3%) was markedly higher than that of CD (21.4%, Table 1). In Japan, the incidence of adrenocortical adenoma-induced CS and the incidence of CD were 50.9% and 37.9%, respectively [13]. The distribution of adrenocortical adenoma-induced CS and pituitary CD is different between Caucasian (7.8–18.8% for adenoma-induced CS and 60–80% for pituitary CD) and Asian (50.9–58.3% and 21.4–37.9%, resp.) populations [6, 7, 14, 15]. The incidence of adrenocortical adenoma and CD by gender distribution is higher amongst females (Table 1). This differential distribution of CS incidence by gender has been noted in other published articles [14, 15].

Elevated baseline 24 h UFC levels, an absence of normal plasma cortisol circadian rhythm, and loss of normal suppression of cortisol secretion by LDDST are all highly sensitive tests for the detection of CS [1, 6, 15, 16]. The upper normal range for baseline UFC values in most assays is between 80 and 120 *μ*g/24 h [2]. The determination of the 24 h UFC value is the most sensitive (95 to 100%) and specific (98%) screening test for CS if the collection of urine is adequate as documented by creatinine excretion [5, 7]. One meta-analysis of 15 separate studies found that baseline UFC measurement had a sensitivity of 96.6% and a specificity of 94.3% in the diagnosis of CS [17]. A survey of 146 patients with CS also reported a sensitivity of 95% but also noted that 11% of their patients had at least one out of four UFC measurements within the reference range [17]. Thus, CS cannot be excluded on the basis of a single normal baseline UFC value [15]. Multiple UFC measurements are needed because even intelligent and carefully instructed patients can make mistakes, such as not discarding the urine voided when the first day's collection begins. Furthermore, cortisol excretion in patients with CS may vary from day to day and can be cyclical, so multiple values are necessary to indicate the constancy of cortisol secretion [6]. Moreover, if there is renal impairment with a glomerular filtration rate of less than 30.0 mL/min, or an incomplete collection (such as the urine voided to the toilet during bowel movement), the UFC concentration may be falsely low [3, 18]. Milder elevations of UFC levels can be found in conditions such as chronic anxiety, depression, and alcoholism, all of which are known as pseudo-Cushing states, as well as in normal pregnant women [3]. In this study, the sensitivity of baseline 24 h UFC values using a cut-off value greater than 122 or 80 *μ*g/24 h was 88.5% or 96.2%, respectively, in the all patients with CS (Table 2). Alexandraki and Grossman [19] reported that the baseline UFC was almost total overlap between CS and pseudo-Cushing groups. A value of UFC greater 100 nmol/L (3.6 *μ*g/dL) on the second day of a 2-day LDDST produced a specificity of 100% and a sensitivity of only 56% for the diagnosis of CS versus pseudo-Cushing, whereas a 48 h plasma cortisol of at least 38 nmol/L (1.4 *μ*g/dL) gave a specificity of 100% and a sensitivity of 90%. Since the sensitivity and specificity of UFC were less than ideal when compared with other diagnostic modalities, they suggested the use of other more novel tests as first-step diagnostic tests to screen for hypercortisolemia.

Patients in our study often exhibited baseline morning plasma cortisol levels within the normal range (60%, 48 out of 80 cases) but most had lost circadian rhythm (95.9%, Table 2). In an Italian multicenter study [15], 37% of the patients with CS had a normal baseline plasma cortisol but 90% lacked circadian rhythm and 95% lacked suppression after overnight LDDST. Newell-Price et al. [20] reported that a single sleeping midnight cortisol value greater than 1.8 *μ*g/dL resulted in 100% sensitivity for the diagnosis of CS and no overlap with controls. However, this test requires that the patient be admitted for a period of 48 h or more to avoid false-positive responses due to the stress of hospitalization. Likewise, blood samples need to be drawn within 5 minutes of waking the patient. To avoid false positive results due to anticipation-related stress, these patients should not be informed that the test is to be performed [20]. In the Giraldi et al. study [21], the specificity was very low (20.2%) with a midnight plasma cortisol cut-off value of 1.8 *μ*g/dL; specificity increased to 73.9% and 87.7% with 5 *μ*g/dL and 7.5 *μ*g/dL cut-off values, respectively. Regardless, the measurement of midnight plasma cortisol levels is inconvenient and impractical for outpatients. Our study set an easy, practical cut-off point that correlates with loss of plasma cortisol circadian rhythm (i.e., a baseline plasma cortisol level greater than 7.5 *μ*g/dL at 1600–2200 h and greater than 50% of baseline plasma cortisol level at 0800–0900 h) and had a high sensitivity (95.9%) for all patients with CS (Table 2).

The LDDST is used to differentiate CS patients from those who do not have CS [3]. Invitti et al. found the sensitivity to be 95% for the overnight LDDST using the criterion for nonsuppression of serum cortisol greater than 5 *μ*g/dL [15]. The overnight LDDST with a cut-off value of plasma cortisol levels greater than 1.8 *μ*g/dL allowed adequate sensitivity (98%–100%) but low specificity [4, 7]. With a cut-off point using the traditional plasma cortisol level of greater than 5 *μ*g/dL, the overnight LDDST has been reported to have a specificity of 77% to 99% depending on the population studied. Lowering this cut-off would likely further decrease specificity, resulting in an increased number of false positives and potentially unnecessary evaluations. A test with near 100% sensitivity may not be a useful test if its specificity is unacceptably low [4]. Conversely, one review article reported that 3–8% of patients with CD retain sensitivity to dexamethasone and show suppression of plasma cortisol to less than 1.8 *μ*g/dL on overnight or 2-day LDDST. Thus, if clinical suspicion of CS remains high, repeated tests and other investigations are indicated [18]. Findling et al. [1] showed that 18% of patients with CD had plasma cortisol levels suppressed to less than 5 *μ*g/dL (false negative) after overnight LDDST, whereas 8% actually showed suppression of plasma cortisol to less than 2 *μ*g/dL. In addition, they reported that the 2-day LDDST yielded false negative results in 38% of patients when urine cortisol was tested. They recommended that neither the overnight LDDST nor the 2-day LDDST should be used as the sole criterion to exclude the diagnosis of CS [1].

The HDDST relies on the concept that pituitary corticotroph tumor cells in CD retain sensitivity to negative feedback effects of glucocorticoid, but this sensitivity is not observed in EAS or adrenal CS [3, 5, 8, 14]. The 2-day HDDST in our study for the differential diagnosis of adrenal CS demonstrated a sensitivity of 95.5% using the criterion of a cut-off value of plasma cortisol levels greater than 50% of the baseline plasma cortisol level (Table 2). This test demonstrated a sensitivity of 88.9% when the criterion of a cut-off value of the 24 h UFC level greater than 50% of the baseline 24 h UFC level was used. The 2-day HDDST also demonstrated a sensitivity of 44.4% when using the criterion greater than 50% decrease of baseline plasma cortisol levels and a sensitivity of 85.7% when using the criterion greater than 50% decrease of the baseline 24 h UFC value for the differential diagnosis of CD (Table 2). Both overnight HDDST and the 2-day HDDST can distinguish pituitary from ectopic source of ACTH with a sensitivity varying from 60% to 80% and a high specificity when a cut-off value of plasma cortisol suppression above 50% is used [3]. The 2-day HDDST reportedly carries a sensitivity of 89–94% and a widely ranging specificity (from 29–60%) using the criterion of a 50% decrease in UFC levels. The overnight HDDST bears a sensitivity of 77–92% and a specificity of 57–100% using the criterion of a 50% decrease in the plasma cortisol levels for the differential diagnosis of ACTH-dependent CS [15]. Isidori et al. [22] found that 7 of 36 patients with EAS (19%) are suppressed to more than 50% baseline cortisol levels during the HDDST. They also found that more than 60% suppression of baseline cortisol levels had the highest accuracy in excluding EAS, with 80% sensitivity and 90% specificity for the diagnosis of CD. The combined criteria of a more than 30% suppression of serum cortisol during the LDDST and/or a more than 20% increase in the CRH test had a significantly higher sensitivity (97%) and specificity (94%) than the HDDST or the CRH tests alone in the differential of ACTH-dependent CS. Factors that influence the poor diagnostic accuracy of the HDDST include incomplete urine collection, interfering substances, episodic cortisol secretion, or inadequate levels of plasma dexamethasone due to inadequate absorption, increased clearance, or poor compliance [8, 14]. As a result, the HDDST should not be used as the sole test for the differential diagnosis of ACTH-dependent CS [8].

The determination of a baseline plasma ACTH level is the best way to discriminate between ACTH-independent CS and ACTH-dependent CS [5]. ACTH concentrations below the level of detection or 10 pg/mL at 0900 h with concomitant increased production of cortisol suggest an ACTH-independent cause of CS. Plasma ACTH values greater than 20 pg/mL suggest an ACTH-dependent cause. For values between 10 and 20 pg/mL, a CRH stimulation test is indicated [3]. One retrospective review examined over 425 patients with CS and determined that 28% of patients with ACTH-independent CS had ACTH levels in the normal range. In addition, eight patients with adrenal adenomas had ACTH levels of 20 pg/mL or greater [4, 15]. Our study showed no overlap of baseline plasma ACTH levels between ACTH-independent CS and ACTH-dependent CS. Five patients (11.6%) with adrenal CS had ACTH levels above 20 pg/mL (Table 3). Invitti et al. reported that CRH test was particularly useful for the exclusion of a pituitary adenoma in patients with ACTH-independent CS and normal ACTH levels, because ACTH did not respond to CRH in any of these patients [15]. On the other hand, two-day or overnight HDDST may be considered for distinguishing adrenal CS from CD in cases of plasma ACTH levels below 30 pg/L. Patients with nonsuppressible plasma cortisol levels during the HDDST favor adrenal CS. 

Laparoscopic adrenalectomy has become a common procedure for most benign functioning and nonfunctioning adrenal masses, as well as for patients with hyperplasia [23]. The advantages of laparoscopic adrenalectomy as compared with open adrenalectomy include briefer postoperative hospital stay, less postoperative pain and complications, earlier oral feeding, quicker mobilization, and better postoperative quality of life and cosmetic results [23, 24]. However, Gonzalez et al. [25] reported an association between laparoscopic adrenalectomy for adrenocortical carcinoma and a high risk of peritoneal carcinomatosis (83%) compared with open adrenalectomy (8%, *P* = 0.0001). These researchers suggested that open adrenalectomy should remain the standard of care for resection of adrenal cortical neoplasms for which adrenocortical carcinoma remains in the differential diagnosis. Such tumors include those with imaging characteristics suggestive of malignancy (e.g., heterogeneous or irregular borders) or a transverse diameter greater than 4 cm.

## 5. Conclusions

The most common cause of CS in our study was adrenal dependent. In the definitive diagnosis of all the CS patients, loss of plasma cortisol circadian rhythm, baseline 24 h UFC values >80 *μ*g/dL, and overnight and 2-day LDDST exhibited a high sensitivity (94.4–100%). Multiple UFC measurements and repeated LDDST are needed if clinical suspicion of CS remains high. In the differential diagnosis of CS, baseline plasma ACTH concentration is the best test to discriminate between ACTH-independent CS and ACTH-dependent CS. The HDDST should not be used as the sole test for the differential diagnosis of ACTH-dependent CS. Open adrenalectomy should be reserved for cases in which adrenocortical carcinoma remains in the differential diagnosis.

## Figures and Tables

**Table 1:** Patient distribution and demographic data for the 84 patients with various causes of Cushing's syndrome.

Causes of Cushing's syndrome	Number of patients (%)	Sex (F : M)	Mean age (yr) at operation (range)
ACTH-independent Cushing's syndrome	63 (75%)	46 : 17	37.5 ± 14.2 (0.7–67.7)
(1) Adrenocortical adenoma	49 (58.3%)	40 : 9	35.3 ± 13.1 (0.7–67.7)
Unilateral	48 (57.1%)	39 : 9	
Bilateral	1 (1.2%)	1 : 0	
(2) Adrenocortical carcinoma	7 (8.3%)	5 : 2	44.8 ± 21.8 (1.3–66)
Unilateral	6 (7.1%)	5 : 1	
Bilateral	1 (1.2%)	0 : 1	
(3) PPNAD*	4 (4.8%)	1 : 3	37.8 ± 8.2 (25–45.8)
(4) AIMAH**	3 (3.6%)	0 : 3	54.9 ± 9.8 (43.8–62.6)
ACTH-dependent Cushing's syndrome	21 (25%)	16 : 5	40.9 ± 16.1 (14–67)
(1) Cushing's disease	18 (21.4%)	15 : 3	40.3 ± 16.3 (14–67)
(2) Ectopic ACTH syndrome	3 (3.6%)	1 : 2	45.1 ± 17.5 (25–57)

*PPNAD: primary pigmented nodular adrenocortical disease.

**AIMAH: ACTH-independent bilateral macronodular adrenocortical hyperplasia.

**Table 2:** Test results for ACTH-independent CS and ACTH-dependent Cushing's syndrome (CS) in the 84 patients studied.

Tests	ACTH-independent CS% (number of patients)	ACTH-dependent CS% (number of patients)	All patients with CS% (number of patients)	Cushing's disease% (number of patients)	Ectopic CS% (number of patients)
Loss of cortisol circadian					
rhythm (+)*	96.4% (53/55)	94.4% (17/18)	95.9% (70/73)		
Baseline plasma cortisol levels					
at 1600–2200 h > 7.5 *μ*g/dL	98.3% (58/59)	94.4% (17/18)	97.4% (75/77)		
Baseline 24 h UFC					
>122 *μ*g/24 h	87.5% (35/40)	91.7% (11/12)	88.5% (46/52)		
>80 *μ*g/24 h	97.5% (39/40)	91.7% (11/12)	96.2% (50/52)		
Overnight LDDST**					
0800 h plasma cortisol > 5 *μ*g/dL: nonsuppression	100% (48/48)	88.9% (8/9)	98.2% (56/57)	85.7% (6/7)	100% (2/2)
2-day LDDST^§^					
0800 h plasma cortisol > 5 *μ*g/dL: nonsuppression	100% (25/25)	100% (7/7)	100% (32/32)	100% (5/5)	100% (2/2)
24 h UFC > 122 *μ*g/day: nonsuppression	100% (15/15)	66.7% (2/3)	94.4% (17/18)	50% (1/2)	100% (1/1)
2-day HHDST^§§^					
0800 h plasma cortisol					
Nonsuppression^†^	95.5% (42/44)			55.6% (5/9)	100% (2/2)
Suppression	4.5% (2/44)			44.4% (4/9)	
24 h UFC					
Nonsuppression^‡^	88.9% (24/27)			14.3% (1/7)	100% (1/1)
Suppression	11.5% (3/26)			85.7% (6/7)	

*Loss of cortisol circadian rhythm (+): baseline plasma cortisol levels at 1600–2200 h greater than 7.5 *μ*g/dL and greater than 50% of baseline plasma cortisol levels at 0800–0900 h.

**Overnight LDDST: overnight low-dose dexamethasone suppression test: dexamethasone 1 mg orally at 2300 h and blood sampling for cortisol the following morning at 0800 h.

^§^2-day LDDST: 2-day low-dose dexamethasone suppression test: dexamethasone 0.5 mg orally every 6 hours for a total of 8 doses and blood sampling for cortisol at 0800 h on the third day of testing. Collection of urine for free cortisol for 24 h from 0800 h on the second day to 0800 h on third day following dexamethasone administration.

^§§^2-day HDDST: 2-day high-dose dexamethasone suppression test: dexamethasone 2 mg orally every 6 hours for a total of 8 doses and blood sampling for cortisol at 0800 h on the third day of testing. Collection of urine for free cortisol for 24 h from 0800 h on the second day to 0800 h on the third day following dexamethasone administration.

^†^Nonsuppression of 0800 h plasma cortisol on 2-day HDDST: a cut-off value of 0800 h plasma cortisol after 2-day HDDST is greater than 50% of baseline plasma cortisol.

^‡^Nonsuppression of 24 h urine free cortisol on 2-day HDDST: a cut-off value of 24 h urinary free cortisol after 2-day HDDST is greater than 50% of baseline 24 h urinary free cortisol.

**Table 3:** Comparison of baseline plasma ACTH levels between patients with ACTH-independent Cushing's syndrome and ACTH-dependent Cushing's syndrome (CS).

Baseline plasma ACTH levels (pg/mL) at 0800 h	ACTH-independent CS% (number of patients)	Cushing's disease% (number of patients)	Ectopic CS% (number of patients)
0 < ACTH ≦ 10	62.8% (27/43)		
10 < ACTH ≦ 20	25.6% (11/43)		
20 < ACTH ≦ 28.7	11.6% (5/43)		
37.4 ≦ ACTH ≦ 52		18.8% (3/16)	
52 < ACTH ≦ 328		81.3% (13/16)	33.3% (1/3)
427 ≦ ACTH ≦ 725			66.7% (2/3)

Normal range of baseline plasma ACTH levels: 9–52 pg/mL.
